# The Treatment of Neuralgia by Electrization

**Published:** 1869-03

**Authors:** A. D. Rockwell, G. M. Beard

**Affiliations:** Lecturer in the Medical Department of the University of New York


### Selections.

THE TREATMENT OF NEURALGIA BY ELECTRIZATION.—By A. D. ROCKWELL, M. D., AND G. M. BEARD, M. D., *Lecturer in the Medical Department of the University of New York.*—Under the general term neuralgia, which, fifty years ago, was but little known, either to the profession or the laity, is now included one of the most frequent and distressing symptoms of the chronic diseases of our time.

The term neuralgia (neuron and algas—pain of the nerve) gives no clue whatever to the nature of the affection.

Strictly speaking, all pain, in any disease, is nerve pain, and therefore the term neuralgia might be applied to every phase of disease, acute or chronic, that is attended with unpleasant sensations. This term, however, as ordinarily employed, designates an affection of the nervous system, which is attended with pain in the course of some of the principal sensory nerves.

The pain of most acute and many chronic diseases, is diffused through the various tissues, either locally as in arthritis, abscess or other swelling, or generally as in constitutional rheumatism and other febrile affections.

When, in any disease, the pain follows the course of any particular or prominent nerve-branch, it receives the name neuralgia. The pains of the affection are usually quite sudden in their onset, and are of a lacerating, stabbing, darting, or burning character. They are more or less intermittent, and are not ordinarily accompanied by any constitutional febrile disturbance.

Neuralgia has usually been classified according to the locality of the pain, and corresponding special names are given to it. Thus we have facial, brachial, intercostal, and abdominal neuralgia; gastralgia, sciatica, etc. This method of classification, though convenient, and to a certain extent indispensable, is yet, to the last degree, unphilosophical and unscientific. It grew up in the times of professional ignorance, and therefore gives no idea of the special nature of the affection, and can be no guide in the prognosis or therapeutics. The method of classification of the neuralgias that we adopt is *based on the local or general condition in which they take their origin.*

It seems to us at once logical, scientific, and readily suggestive, both of the prognosis and the therapeutics. All conceivable forms and phases of this affection may be included under one or the other of these four grand divisions:

1st. *Constitutional Neuralgias.*—Those which arise from constitutional conditions: anæmia, neurasthenia (nervous exhaustion), poisoning by syphilis, mercury, rheumatism, gout.

2d. *Central Neuralgias.*—Those which arise from disease of the central nervous system: inflammation, congestion, anæmia, exhaustion of the brain and spinal cord, also meningitis, tumors, pressure of foreign substances, etc.

3d. *Peripheric Neuralgias.*—Those which arise from local diseases of, injury to, or pressure on the nerve; neuritis, neuroma, aneurisms, wounds, bruises, etc.

4th. *Reflex Neuralgias.*—Those which arise from reflex action. This class embraces a large number of neuralgias that attack all portions of the body. Complicated cases occur that may properly belong to two or more of these divisions. A patient afflicted with anæmia or neurasthenia may suffer from neuralgia that may be aggravated by neuritis, or by a wound or bruise. A curable case of neuralgia of malarial origin, may be rendered incurable by the supervention of organic disease of the brain or spinal cord. Illustrations of these varied forms are sufficiently familiar to the practitioner. The prognosis of the affection manifestly depends on its causation. It is impossible to give an intelligent opinion in any given case, without first ascertaining the predominant condition on which the symptoms depend. The principles on which neuralgia is to be treated, are simply these two: *first, to relieve the pain*, and *secondly, to remove the cause.*

The relief of pain is accorded the preference, because it is the most urgent, on account of its terrible severity. The ordinary methods of fulfilling these two indications we do not propose to discuss, but shall confine ourselves to the treatment by electricity, in the form of general, partial, and localized electrization with the faradaic and galvanic currents.

The treatment of neuralgia by the electric currents has occupied the attention of the more advanced neurologists and electricians for a number of years. Meyer devotes a small portion of his monograph to this subject.

The current from the voltaic pile was recommended for neuralgia by Grapengressen at the beginning of the present century. The galvanic current from a number of elements was employed by the late Prof. Remak, and by him this method of treatment was introduced to the profession of our time.

Rosenthal devotes a very interesting chapter to this theme, and relates a number of suggestive cases.

Althaus speaks of neuralgia in both of his works on electro-therapeutics, and records very satisfactory results.

Benedict, whose recently published treatise on electro-therapeutics is incomparably superior to any other work on this subject, in any language, enters into the consideration of the treatment of neuralgia with elaborate and scientific detail, and reports a large variety of cases.

All of these writers have achieved their results by the use of *localized* electrization merely, since this is the method which is chiefly employed in Europe.

For reasons that will be presented further on, which will, we think, commend themselves to the judgment, and accord with experience, *general electrization* is oftentimes far more efficacious than localized, especially in those cases of neuralgia that depend on constitutional conditions.

Electrization, wisely administered, fulfills, to a greater or less degree, both of the conditions that are required in the treatment of neuralgia. It oftentimes relieves pain and cures the cause of it. When applied all over the person, from the head to the feet, it acts as a powerful constitutional tonic, and *thus* helps to remove very frequent and persistent causes of neuralgia.

In order that treatment by electricity may be successful, there are needed apparatus for both currents, the galvanic and the faradaic. For the faradaic current we use the apparatus of Kidder. For the galvanic we now employ to a considerable extent, the portable air-tight galvanic apparatus of the Messrs. Chester. The general rules that should guide us in the treatment of neuralgia by electricity are these:

1st. All forms, constitutionals, central, peripheric and reflex, that are dependent on, or associated with general debility, should be treated by general electrization, and *usually* with the faradaic current.

2d. Acute and sub-acute neuralgias, of a merely local character, are *usually* best treated by a very mild galvanic current, *steadily* applied over the painful nerves.

These forms of neuralgia, however, sometimes yield to the faradaic current.

